# Preventive Effects of Pentoxifylline on the Development of Colonic Premalignant Lesions in Obese and Diabetic Mice

**DOI:** 10.3390/ijms18020413

**Published:** 2017-02-15

**Authors:** Kazufumi Fukuta, Yohei Shirakami, Akinori Maruta, Koki Obara, Soichi Iritani, Nobuhiko Nakamura, Takahiro Kochi, Masaya Kubota, Hiroyasu Sakai, Takuji Tanaka, Masahito Shimizu

**Affiliations:** 1 Department of Gastroenterology, Gifu University Graduate School of Medicine, Gifu 501-1194, Japan; kazufumi19780802@yahoo.co.jp (K.F.); mrak5844@yahoo.co.jp (A.M.); silent_jealousy0308@yahoo.co.jp (K.O.); is590124@yahoo.co.jp (S.I.); xenon2112@gmail.com (N.N.); kottii924@yahoo.co.jp (T.K.); kubota-gif@umin.ac.jp (M.K.); sakaih03@gifu-u.ac.jp (H.S.); shimim-gif@umin.ac.jp (M.S.); 2 Department of Informative Clinical Medicine, Gifu University Graduate School of Medicine, Gifu 501-1194, Japan; 3 Department of Pathological Diagnosis, Gifu Municipal Hospital, Gifu 500-8513, Japan; tmntt08@gmail.com

**Keywords:** colorectal cancer, pentoxifylline, chemoprevention, obesity, oxidative stress, inflammation

## Abstract

Obesity and its related metabolic abnormalities, including enhanced oxidative stress and chronic inflammation, are closely related to colorectal tumorigenesis. Pentoxifylline (PTX), a methylxanthine derivative, has been reported to suppress the production of tumor necrosis factor (TNF)-α and possess anti-inflammatory properties. The present study investigated the effects of PTX on the development of carcinogen-induced colorectal premalignant lesions in obese and diabetic mice. Male C57BL/KsJ-*db*/*db* mice, which are severely obese and diabetic, were administered weekly subcutaneous injections of the colonic carcinogen azoxymethane (15 mg/kg body weight) for four weeks and then received drinking water containing 125 or 500 ppm PTX for eight weeks. At the time of sacrifice, PTX administration markedly suppressed the development of premalignant lesions in the colorectum. The levels of oxidative stress markers were significantly decreased in the PTX-treated group compared with those in the untreated control group. In PTX-administered mice, the mRNA expression levels of cyclooxygenase (COX)-2, interleukin (IL)-6, and TNF-α, and the number of proliferating cell nuclear antigen (PCNA)-positive cells in the colonic mucosa, were significantly reduced. These observations suggest that PTX attenuated chronic inflammation and oxidative stress, and prevented the development of colonic tumorigenesis in an obesity-related colon cancer model.

## 1. Introduction

The increasing worldwide prevalence of obesity presents a serious health issue owing to the elevated risk of medical problems, including diabetes mellitus, ischemic heart disease, stroke, and various types of cancers [1,2]. In particular, the risk of colorectal cancer (CRC) is known to be particularly high in individuals with obesity [3,4]. Therefore, in addition to early detection and treatment, the importance of preventive action, including the improvement of lifestyle habits and utilization of chemopreventive agents such as nonsteroidal anti-inflammatory drugs or aspirin, is recognized in the management of CRC [5,6,7].

The mechanisms by which obesity and diabetes promote the development of CRC have been partly elucidated and include insulin resistance, adipokine imbalance, oxidative stress, and chronic inflammation [3,4,8,9,10]. Previous reports have indicated that chemically induced colorectal carcinogenesis is enhanced in obese and diabetic mice [11], and mice with diet-induced obesity were markedly susceptible to the development of colon tumors [12]. Meanwhile, recent investigations have reported that several types of natural compounds, such as green tea catechin and curcumin, inhibited the development of obesity-related colorectal tumorigenesis through the attenuation of chronic inflammation [13,14]. In addition, administration of the xanthophyll carotenoid astaxanthin, and an angiotensin-converting enzyme inhibitor, also suppressed the early phase of colorectal carcinogenesis in experimental obese rodents by the attenuation of inflammation and oxidative stress [15]. These reports suggested that targeting obesity-related metabolic abnormalities such as chronic inflammation and oxidative stress was a promising strategy for the prevention of CRC in obese individuals [4].

The methylxanthine derivative pentoxifylline (PTX), which works as a competitive non-selective phosphodiesterase inhibitor, is a medicinal agent used to ameliorate circulation in peripheral vascular disorders [16,17]. A recent paper reported that PTX ameliorated the histopathological appearance of non-alcoholic steatohepatitis (NASH), which is closely associated with obesity and metabolic syndromes, in a randomized placebo-controlled trial [18]. In a mouse model, PTX also prevented NASH-related liver tumorigenesis through the attenuation of chronic hepatic inflammation [19]. In addition, PTX has been reported to suppress tumor necrosis factor (TNF)-α synthesis and oxidative stress and improve the pathophysiological conditions of chronic inflammatory diseases [20,21]. Therefore, we expected that PTX, which appears to have anti-inflammatory properties, might be able to attenuate chronic inflammation induced by obesity and to suppress their associated colon carcinogenesis.

A useful preclinical rodent model has been developed in C57BL/KsJ-*db*/*db* (*db*/*db*) mice, which have a leptin receptor mutation and display hyperphagic obesity and diabetes [22], after the injection of a colonic carcinogen azoxymethane (AOM). This appears to be a feasible model to investigate obesity-related colorectal carcinogenesis [14,23,24,25]. In the present study, we investigated the effects of PTX on the development of premalignant lesions in the mouse model of obesity-related and AOM-induced colorectal carcinogenesis.

## 2. Results

### 2.1. General Observations

As shown in Table 1, there was no significant difference in body weight in all three groups at the termination of the experiment. Significant differences were also not observed in the mean weights of the liver, kidney, and fat between the groups. Histopathological examination revealed that PTX was not toxic to mice tissues. Administration with PTX had no effect on the colon length.

### 2.2. Pentoxifylline (PTX) Affects Azoxymethane (AOM)-Induced β-Catenin Accumulated Crypts (BCAC) and Aberrant Crypt Foci (ACF) Formation in db/db Mice

**Figure 1:**
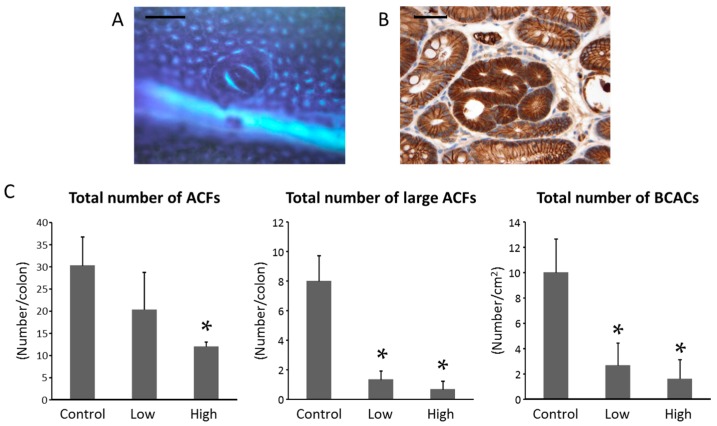
Azoxymethane (AOM)-induced colonic preneoplastic lesions aberrant crypt foci (ACF) and β-catenin accumulated crypts (BCACs) in male C57BL/KsJ-*db/db* mice. Representative pictures of AOM-induced colonic preneoplastic lesions; (**A**) ACF revealed by methylene blue staining and (**B**) BCACs stained immunohistochemically for β-catenin. Scale bars, 200 µm (left); 50 µm (right); (**C**) The numbers of ACF and BCACs observed in each group. Large ACFs, ACFs with four or more aberrant crypts. Each column represents the mean ± SD (*n* = 6 for each group). * *p* < 0.05 vs. pentoxifylline (PTX)-untreated control group.

Colorectal premalignant lesions, aberrant crypt foci (ACF, Figure 1A), and β-catenin accumulated crypts (BCACs, Figure 1B) [26,27] developed in the colons of all mice in the study. Figure 1C displays the number of ACF and BCACs observed in each group. In comparison to the PTX-untreated control group, treatment with a high-dose of PTX significantly reduced the number of ACF (*p* < 0.05). In addition, the number of large ACF, which consist of more than four aberrant crypts and possess greater tumorigenic potential [28], was significantly smaller in both the low- and high-dose PTX-treated groups than that of the PTX-untreated control group (*p* < 0.05). Analysis of the total number of BCACs per unit area revealed that the values in both the low- and high-dose PTX-treated groups were also significantly lower than that of the PTX-untreated group (*p* < 0.05). When comparing between the low- and high-dose PTX-treated groups, there was no statistically significant difference in the number of ACF, large ACF, or BCAC.

### 2.3. PTX Affects Cell Proliferation in Colonic Mucosa of Experimental Mice

**Figure 2:**
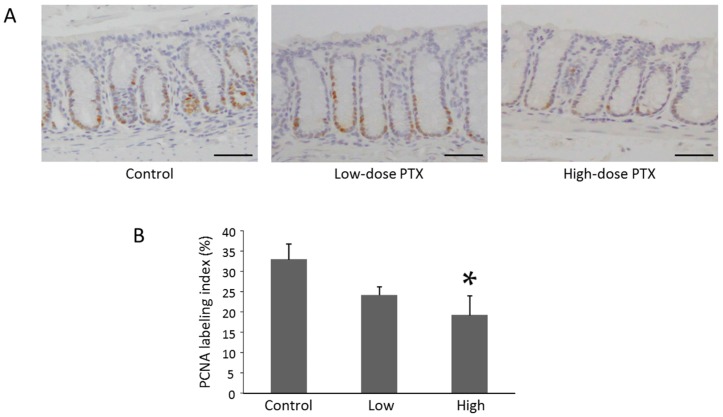
Effects of PTX on cellular proliferation of colon epithelium in the experimental mice. (**A**) Assessment of the normal crypts in the colon of AOM-treated *db*/*db* mice using antibody for proliferating cell nuclear antigen (PCNA). Sections of the colon from each group were stained immunohistochemically with anti-PCNA antibody, as described in the Materials and Methods section. Representative photomicrographs from each group are shown. Scale bars, 200 µm; (**B**) Evaluation of PCNA labeling index in the normal crypts in the colon of the experimental mice. Each column represents the mean ± SD (*n* = 6 for each group). * *p* < 0.05 vs. PTX-untreated control group.

Treatment with PTX, especially in the high-dose group, significantly decreased the proliferating cell nuclear antigen (PCNA)-labeling indices of non-lesional crypts (*p* < 0.05) (Figure 2). This observation indicated that PTX significantly suppressed cell proliferation in the colonic mucosa of AOM-treated *db*/*db* obese mice.

### 2.4. PTX Affects Systemic Oxidative Stress of Experimental Mice

**Figure 3:**
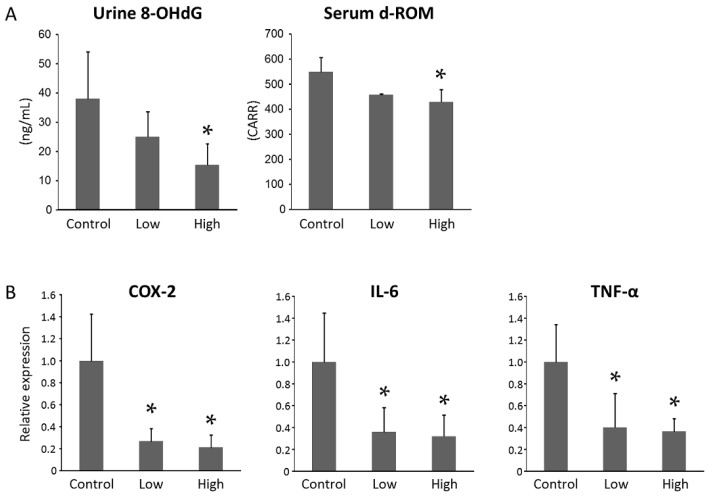
Oxidative stress and expression levels of genes related to inflammation in the colonic mucosa of experimental mice. (**A**) Measurement of urine 8-hydroxy-2′-deoxyguanosine (8-OHdG) and serum derivatives of reactive oxygen metabolites (d-ROMs) levels (Ctrl, *n* = 9; Low, *n* = 11; High, *n* = 10); (**B**) The mRNA expression levels of cyclooxygenase (COX)-2, interleukin (IL)-6, and tumor necrosis factor (TNF)-α in the colonic mucosa were measured by quantitative real-time reverse transcription (RT)-PCR with specific primers (*n* = 6 for each group). Triplicate experiments were performed. Each column represents the mean ± SD. * *p* < 0.05.

As oxidative stress is implicated in obesity-related colorectal carcinogenesis [8], the effect of PTX treatment on oxidative stress levels in the experimental mice was investigated. To achieve this, the levels of oxidative stress markers, such as urinary 8-hydroxy-2′-deoxyguanosine (8-OHdG) and serum derivatives of reactive oxygen metabolites (d-ROMs), both of which are increased by AOM-treatment [29], were examined. As shown in Figure 3A, the level of urinary 8-OHdG, which reflects DNA damage induced by oxidative stress, was significantly decreased by administration of a high-dose of PTX (*p* < 0.05). Treatment with a high-dose of PTX also reduced serum d-ROMs, which are a marker for hydroperoxide levels (*p* < 0.05). When comparing between the low- and high-dose PTX-treated groups, there was no statistically significant difference in the markers for oxidative stress.

### 2.5. Effects of PTX on mRNA Levels of Cyclooxygenase (COX)-2, Arginase, Lipoxygenase, and Inflammatory Cytokines in Colonic Mucosa of AOM-Injected db/db Mice

Real-time reverse transcription (RT)-PCR analyses revealed that both a high- and low-dose of PTX treatment markedly reduced the mRNA expression levels of cyclooxygenase (COX)-2, which is an important mediator in the inflammatory pathway and involved in the development of CRC [30], compared with the control mice (Figure 3B, *p* < 0.05). The levels of pro-inflammatory cytokines interleukin (IL)-6 and TNF-α in the colonic mucosa of PTX-treated mice were also significantly decreased in comparison with those in the control mice (*p* < 0.05). When comparing between the low- and high-dose PTX-treated groups, there was no statistically significant difference in the levels of COX-2, IL-6, and TNF-α. Other inflammatory cytokines and mediators which are related to pathways in the early steps of CRC development, including arginase-1, IL-23a, IL-27, 12-lipoxygenase (LOX), and 15-LOX in the colonic mucosa, were examined as well, demonstrating that they showed no significant difference of expression levels among the three groups (Figure S1A).

### 2.6. PTX Did Not Affect Serum Parameters in Experimental Mice

Many obesity-associated complications, including diabetes and dyslipidemia, are known to be involved in colorectal tumorigenesis [3,4,8] and PTX has been shown to exert beneficial effects on glucose metabolism and insulin resistance in patients with NASH and diabetes [31]. Therefore, serum parameters related to these metabolic disorders were evaluated. Following the administration of PTX, no changes in metabolic parameters, including free fatty acids, total cholesterol, triglycerides, glucose, insulin, and the indices of homeostasis model assessment of insulin resistance (HOMA-R) and quantitative insulin sensitivity check index (QUICKI), were observed at the end of the study (Table 2). The serum concentration of TNF-α was also measured using an enzyme immunoassay, which was not affected by the treatment with PTX (Figure S1B).

## 3. Discussion

Obesity, a serious health issue worldwide, is a significant risk factor for colorectal carcinogenesis [3,8]. Chronic inflammation and oxidative stress are the key mechanisms connecting obesity and CRC development [9,10]. The present study clearly showed the first evidence that PTX, a non-selective phosphodiesterase inhibitor with antioxidant activity [16,17], markedly suppressed the development of ACF and BCAC, which are both precursor lesions for CRC [26,27] in AOM-treated *db/db* mice. This suppression was presumed to occur through the attenuation of oxidative stress and the reduction of pro-inflammatory cytokines, including TNF-α and IL-6, in the colonic mucosa. In addition, PTX treatment reduced COX-2 expression levels in the colonic mucosa, which might also contribute to the inhibition of the development of colonic premalignant lesions. COX-2 performs critical functions in the growth of tumor cells and may therefore be an important target for chemoprevention of CRC [30].

Chronic inflammation, which is closely related to obesity [32] is a critical element in the pathogenesis of chronic diseases, including the carcinogenesis of various organs. TNF-α is a fundamental tumor promoter in inflammation-associated carcinogenesis [33]; the PTX-induced reduction in the expression levels of TNF-α in the colonic mucosa observed in this study is therefore of importance. This result is consistent with previous studies, which have demonstrated that the reduction in TNF-α following treatment with chemopreventive agents led to the suppression of obesity-related colorectal tumorigenesis [13,34]. Recent studies have also reported that PTX inhibited obesity-related steatohepatitis and the subsequent liver tumorigenesis by the suppression of pro-inflammatory cytokines such as TNF-α [19,35]. These data, together with the results of the present study, strongly suggest that attenuation of chronic inflammation using PTX might be a promising method for the prevention of obesity- and inflammation-related carcinogenesis.

Obesity and chronic inflammation are known to be often accompanied by enhanced oxidative stress, which is represented by the increased generation of reactive oxygen species [36]. These species are derivatives of molecular oxygen, including hydrogen peroxide and superoxide, and are able to cause genetic mutation, leading to the development of cancers [37,38]. In the present study, the levels of oxidative stress markers, including urinary 8-OHdG and serum d-ROMs, were markedly reduced by the administration of PTX in AOM-injected *db*/*db* mice. These results clearly indicated that attenuation of oxidative stress might be crucial for the PTX-induced suppression of colorectal premalignant lesion development in obese mice. Although the mechanism by which PTX attenuated oxidative stress was not uncovered in our study, a previous report indicated that PTX inhibited oxidative stress via upregulation of the expression levels of antioxidant enzymes such as superoxide dismutase and glutathione [39].

## 4. Materials and Methods

### 4.1. Animals, Chemicals, and Diets

Male *db*/*db* mice were obtained from Japan SLC (Shizuoka, Japan). Mice were cared for and maintained at the Gifu University Life Science Research Center (Gifu, Japan) according to the Institutional Animal Care Guidelines. AOM and PTX were obtained from Wako Pure Chemical Co. (Osaka, Japan).

### 4.2. Experimental Procedure

The experiment comprised 30 male *db*/*db* mice and they were fed the basal diet CRF-1 (Oriental Yeast, Tokyo, Japan). From five weeks of age, all mice received a subcutaneous injection of AOM (15 mg/kg body weight) once a week for four weeks and were then randomly divided into three groups. Mice in group 1 (*n* = 9) received no treatment, while mice in groups 2 (*n* = 11) and 3 (*n* = 10) received tap water containing 125 and 500 ppm PTX, respectively, from one week after the final AOM injection until the end of the experiment. At the termination of the study (17 weeks of age), all mice were sacrificed by CO_2_ asphyxiation for histopathological analysis. The experimental procedure was approved by the Committee of the Institutional Animal Experiments of Gifu University (the authorization code 27-4 on 2 April 2015).

### 4.3. Counting of ACF and BCAC

The frequencies of ACF and BCAC were determined according to previously reported procedures [14,24,25]. The resected colons were fixed in 10% buffered formalin for 24 h, the mucosal surfaces were stained with 0.5% methylene blue, and the number of ACF was counted under a microscope. After counting the ACF, the distal part (1 cm from the anus) of the colon was cut, embedded in paraffin, and stained immunohistochemically for β-catenin to identify the BCAC intramucosal lesions.

### 4.4. Histopathological and Immunohistochemical Analyses for β-Catenin and Proliferating Cell Nuclear Antigen (PCNA)

For all experimental groups, formalin-fixed and paraffin-embedded colonic mucosa sections were stained with hematoxylin and eosin for conventional histopathological analysis. Immunohistochemical staining for PCNA and β-catenin were performed using the labeled streptavidin-biotin method (LSAB kit; Dako, Glostrup, Denmark), as previously described [14,24,25]. The primary antibodies for β-catenin and PCNA were obtained from BD Transduction Laboratories (No. 610154; San Jose, CA, USA) and Santa Cruz Biotechnology (sc-7907; Santa Cruz, CA, USA), respectively. PCNA-positive cells in the colonic mucosa were counted and expressed as a percentage of the total number of normal crypt cells. The PCNA labeling index (%) was determined from the assessment of a minimum of 200 crypt cells in each mouse [29].

### 4.5. RNA Extraction and Quantitative Real-Time RT-PCR

The expression levels of the genes COX-2, IL-6, and TNF-α were determined in the colonic mucosa of experimental mice by the performance of quantitative real-time RT-PCR analysis, as previously described [40]. Other inflammatory cytokines and mediators, including arginase-1, IL-23a, IL-27, 12-LOX, and 15-LOX in the colonic mucosa, were examined as well. Colonic mucosa was scraped and purification of RNA from the sample was performed using the RNeasy Mini Kit (QIAGEN, Venlo, The Netherlands). To synthesize cDNA, the High Capacity cDNA Reverse Transcription Kit (Applied Biosystems, Foster City, CA, USA) was utilized. Quantitative real-time RT-PCR was conducted by a LightCycler Nano (Roche Diagnostics, Indianapolis, IN, USA) with FastStart Essential DNA Green Master (Roche Diagnostics). The specific primers used for the amplification of COX-2, IL-6, and TNF-α and glyceraldehyde-3-phosphate dehydrogenase (GAPDH) genes have been previously described [34,41]. Other primers are shown in Table S1. Gene expression levels were normalized to GAPDH expression.

### 4.6. Oxidative Stress Analysis

To investigate systemic oxidative stress, urine 8-OHdG levels were measured using an enzyme-linked immunosorbent assay kit (NIKKEN SEIL, Shizuoka, Japan) in accordance with the manufacturer’s protocol. Serum hydroperoxide levels were also evaluated using d-ROMs test (FREE Carpe Diem, Diacron International s.r.l., Grosseto, Italy) [42].

### 4.7. Clinical Chemistry

Blood samples were collected from the inferior vena cava of the mice at the time of sacrifice (after 8 h of fasting) and were used for the chemical analyses. The serum levels of free fatty acid (Wako Pure Chemical, Osaka, Japan), total cholesterol (Wako Pure Chemical), triglycerides (Wako Pure Chemical), glucose (BioVision Research Products, Mountain View, CA, USA), and insulin (Shibayagi, Gunma, Japan) were determined by enzyme immunoassay in accordance with the manufacturers’ protocols. Insulin resistance and insulin sensitivity were calculated by evaluation of the homeostasis model assessment of HOMA-R and the QUICKI, respectively [43,44]. The serum level of TNF-α was determined by an enzyme immunoassay according to the manufacturer’s protocol (Shibayagi, Gunma, Japan).

### 4.8. Statistical Analyses

The results were presented as the mean ± SD and one-way ANOVA was used to assess the difference among groups. The Tukey–Kramer multiple comparison test was performed to compare each experimental group with the control group. When *p*-value was less than 0.05, the differences were considered statistically significant.

## 5. Conclusions

This study demonstrated the preventive effects of PTX on the early phase of obesity-related colorectal carcinogenesis. As PTX did not improve glucose metabolism and insulin resistance, which are also involved in CRC development [45], we deduced the preventive effects occurred mainly through inhibition of oxidative stress and inflammation in the colonic epithelium. The risk for CRC is increased by obesity and its related metabolic abnormalities; therefore, targeting the abnormalities, such as chronic inflammation and oxidative stress, might be an efficacious prevention strategy for CRC in obese people. PTX appears to be an effective and practical candidate for this purpose, as it is able to attenuate chronic inflammation and oxidative stress and has been used previously in clinical practice without severe adverse reactions [18]. Further studies should be conducted to examine that PTX can be useful in the chemoprevention of colorectal cancer in obese individuals.

## Figures and Tables

**Table 1:** General observations of the experimental mice.

Group Number	PTX	Number of Mice	Body Weight (g)	Relative Weight (g/100 g Body Weight)			Length of Colon (cm)
				Liver	Kidneys	Fat ^a^	
1	-	9	46.6 ± 6.6 ^b^	5.4 ± 0.9	1.2 ± 0.1	4.8 ± 0.8	15.0 ± 3.5
2	125 ppm	11	41.5 ± 6.3	4.5 ± 0.8	1.1 ± 0.1	4.9 ± 0.4	15.0 ± 1.0
3	500 ppm	10	41.4 ± 5.2	4.4 ± 1.1	1.1 ± 0.1	4.9 ± 0.4	15.6 ± 1.0

^a^ White adipose tissue of the peritestis and retroperitoneum; ^b^ Mean ± SD. PTX, pentoxifylline.

**Table 2:** Serum parameters of the experimental mice.

Group Number	1	2	3
PTX	-	125 ppm	500 ppm
Free fatty acid (µEQ/mL)	1009.2 ± 235.3 ^a^	940.8 ± 460.0	723.6 ± 205.2
Total cholesterol (mg/dL)	97.0 ± 17.5	127.2 ± 23.0	111.4 ± 23.3
Triglyceride (mg/dL)	27.2 ± 8.7	25.2 ± 9.9	24.6 ± 4.0
Glucose (mg/dL)	542.0 ± 69.1	324.0 ± 192.5	462.8 ± 162.5
Insulin (µIU/mL)	3.16 ± 1.2	5.0 ± 2.1	6.7 ± 2.3
HOMA-R	4.2 ± 1.5	4.2 ± 3.4	6.7 ± 3.0
QUICKI	0.31 ± 0.02	0.32 ± 0.05	0.30 ± 0.03

^a^ Mean ± SD. HOMA-R, the homeostasis model assessment of insulin resistance; QUICKI, quantitative insulin sensitivity check index.

## Supplementary Materials

Supplementary materials can be found at www.mdpi.com/1422-0067/18/2/413/s1.

## Abbreviations

ACF	Aberrant crypt foci
AOM	Azoxymethane
BCAC	β-Catenin accumulated crypt
COX	Cyclooxygenase
CRC	Colorectal cancer
*db*/*db*	C57BL/KsJ-*db*/*db*
d-ROM	Derivatives of reactive oxygen metabolite
GAPDH	Glyceraldehyde-3-phosphate dehydrogenase
HOMA-R	The homeostasis model assessment of insulin resistance
IL	Interleukin
LOX	Lipoxygenase
NASH	Non-alcoholic steatohepatitis
8-OHdG	8-Hydroxy-2′-deoxyguanosine
PCNA	Proliferating cell nuclear antigen
PTX	Pentoxifylline
QUICKI	Quantitative insulin sensitivity check index
RT-PCR	Reverse transcription-PCR
TNF	Tumor necrosis factor
