# Supplementary material for: Preventive Effects of Pentoxifylline on the Development of Colonic Premalignant Lesions in Obese and Diabetic Mice
Source: Int J Mol Sci. 2017 Feb 15;18(2):413. doi: 10.3390/ijms18020413 (PMC5343947; doi:10.3390/ijms18020413)
Supplement: Supplementary file 1 [file ijms-18-00413-s001.pdf]

Supplementary Materials: Preventive Effects of Pentoxifylline on the Development of Colonic Premalignant Lesions in Obese and Diabetic Mice

Kazufumi Fukuta, Yohei Shirakami, Akinori Maruta, Koki Obara, Soichi Iritani, Nobuhiko Nakamura, Takahiro Kochi, Masaya Kubota, Hiroyasu Sakai, Takuji Tanaka and Masahito Shimizu

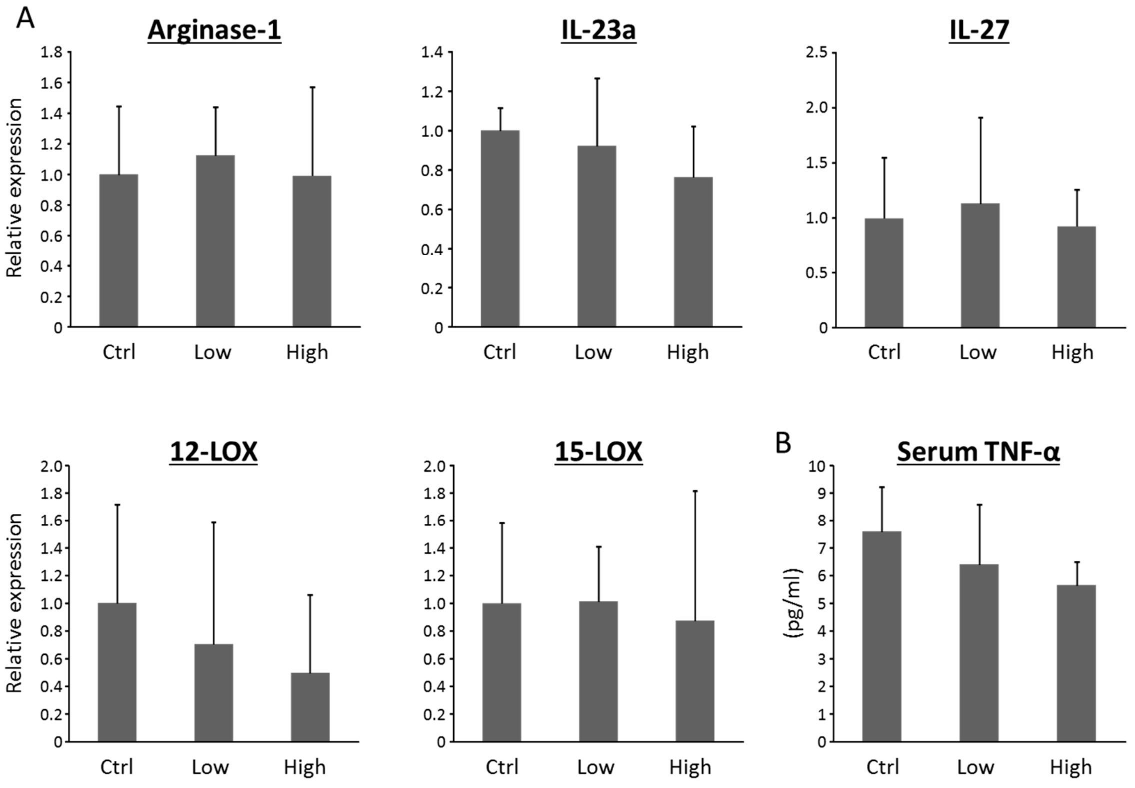

Figure S1. Expression levels of genes related to inflammation in the colonic mucosa and serum level of TNF-α in experimental mice. (**A**) The expression levels of arginase-1, IL-23a, IL-27, 12-LOX, and 15-LOX mRNA in the colonic epithelium were examined by quantitative real-time RT-PCR using specific primers; (**B**) The serum concentrations of TNF-α were measured by an enzyme immunoassay. The values are expressed as mean ± SD.

Table S1. Primer sequences.

Gene	Primer sequences (5′–3′)	
	Forward	Reverse
Arginase-1	GAATCTGCATGGGCAACC	GAATCCTGGTACATCTGGGAAC
IL-23a	CTGTTGCCCTGGGTCACT	AGCCCAGTCAGGACTGCTAC
IL-27	ATGGCATCACCTCTCTGACTC	AAGGGCCGAAGTGTGGTAG
12-LOX	GATCACTGAAGTGGGGCTGT	CACACATGGTGAGGAAATGG
15-LOX	GGGGATGGAGAAGCTACAGG	TCCGCTTCAAACAGAGTGC
